# Blood-nerve barrier disruption and coagulation system activation induced by mechanical compression injury participate in the peripheral sensitization of trigeminal neuralgia

**DOI:** 10.3389/fnmol.2022.1059980

**Published:** 2022-12-20

**Authors:** Lu-Xi Zhou, Shao-Wei Lin, Rong-Hui Qiu, Ling Lin, Yue-Feng Guo, Dao-Shu Luo, Yun-Qing Li, Feng Wang

**Affiliations:** ^1^Laboratory of Clinical Applied Anatomy, Key Laboratory of Brain Aging and Neurodegenerative Diseases, School of Basic Medical Sciences, Fujian Medical University, Fuzhou, Fujian Province, China; ^2^Department of Epidemiology and Health Statistics, Fujian Provincial Key Laboratory of Environment Factors and Cancer, School of Public Health, Fujian Medical University, Fuzhou, Fujian Province, China; ^3^Public Technology Service Center, Fujian Medical University, Fuzhou, Fujian Province, China; ^4^Department of Anatomy, Histology and Embryology & KK Leung Brain Research Centre, Fourth Military Medical University, Xi'an, Shaanxi, China

**Keywords:** mechanical compression, trigeminal root entry zone, blood-nerve barrier, nodes of Ranvier, neurofascin-155, protease-activated receptor 1, trigeminal neuralgia

## Abstract

**Introduction:**

The aim of this study was to investigate the effect and possible mechanisms of the blood-nerve barrier (BNB) and the coagulation-anticoagulation system in modulating the mechanical allodynia in a trigeminal neuralgia (TN) rat model induced by chronic compression of the trigeminal root entry zone (TREZ).

**Methods:**

Von Frey filaments were applied to determine the orofacial mechanical allodynia threshold. The BNB permeability was evaluated by Evans blue extravasation test. Immunohistochemical staining and laser confocal microscopy were used to measure the length of the depletion zones of the nodes of Ranvier in the TREZ, the diameter of nerve fibers and the length of the nodal gap. The transcriptional levels of prothrombin and endogenous thrombin inhibitor protease nexin-1 (PN-1) in the TREZ of TN rats were assessed by RT-qPCR. A Western blotting assay was performed to detect the expression of paranodal proteins neurofascin-155 (NF155) and neurofascin-125 (NF125) in the TREZ. The spatiotemporal expression pattern of thrombin activated receptor (i.e. protease activated receptor 1, PAR1) in TREZ were defined by immunostaining and immunoblotting assays. PAR1 receptor inhibitors SCH79797 were administrated to TN rats to analyze the effect of thrombin-PAR1 on orofacial hyperalgesia.

**Results:**

A compression injury of a rat’s TREZ successfully induced TN-like behavior and was accompanied by the destruction of the permeability of the BNB and the promotion of prothrombin and thrombin inhibitor protease nexin-1 (PN-1) expression. The expression of the paranodal proteins neurofascin-155 (NF155) and neurofascin-125 (NF125) was increased, while the nodal gap length of the nodes of Ranvier was widened and the length of node-depleted zones was shortened. Moreover, the expression of PAR1 within the TREZ was upregulated at an early stage of TN, and administration of the PAR1 antagonist SCH79797 effectively ameliorated orofacial mechanical allodynia.

**Conclusion:**

A compression injury of the TREZ increased the permeability of the BNB and induced disturbances in the local coagulation-anticoagulation system, concomitant with the structural changes in the nodes of Ranvier, thrombin-PAR1 may play a critical role in modulating orofacial mechanical hyperalgesia in a TN rat model.

## Introduction

Primary trigeminal neuralgia (TN) is defined as recurrent severe neuropathic pain in the orofacial area whose pathogenesis is still unknown (Sabalys et al., 2013). The most common etiology of primary TN is compression of the trigeminal root entry zone (TREZ) by a variant artery or vein, and microvascular decompression always effectively relieves TN pain symptoms (Jones et al., 2019). The TREZ is a transitional zone containing central nervous system (CNS) tissues and peripheral nervous system (PNS) tissues in the root of the trigeminal nerve. Astrocytes and myelin sheaths with oligodendrocytes comprise the CNS side of the TREZ, while myelin sheaths formed by Schwann cells comprise the PNS side of the TREZ (Peker et al., 2006; Luo et al., 2019). The transitional zone with different glial interfaces may increase susceptibility to injury-related neural disorders.

It is well known that healthy nodes of Ranvier are crucial for action potential propagation along myelinated axons in both the CNS and PNS. Rapid and efficient action potential conduction depends on the myelin sheath and clustered ion channels at the nodes of Ranvier (Liu et al., 2020). A previous study demonstrated that nearly all (>95%) nodes in the optic nerve, corpus callosum, and spinal cord contain astrocyte processes (Serwanski et al., 2017), which indicates a close relationship between nodes of Ranvier and astrocytes. Both axonal proteins (contactin-associated protein, Caspr) and glial proteins (neurofascin-155, NF155) at paranodes are highly interdependent (D'Este et al., 2017). Caspr is highly expressed on the axon membranes of central and peripheral myelinated nerve fibers at the paranodal region, and NF155 expressed by glial cells connects the myelin sheath to the axon by binding to Caspr. Damage or dysfunction of nodes of Ranvier was reported to contribute to the pathophysiology of various neurological diseases (Susuki, 2013). However, whether orofacial mechanical hyperalgesia in TN is related to abnormal structural changes in nodes of Ranvier in the TREZ remains controversial.

The blood-nerve barrier (BNB) is a peripheral nerve-specific barrier critical for restricting the passive diffusion of potentially harmful soluble mediators and the entry of thrombin from the peripheral blood circulation into the parenchyma of the PNS (Greathouse et al., 2016; Richner et al., 2018). Nerve injury is often accompanied by the destruction of BNB integrity and even participates in the occurrence and development of neuropathic pain (Ubogu, 2020).

Thrombin can be released at high levels following blood vessel damage or tissue injury and can activate protease-activated receptors (PARs; Vellani et al., 2010). Protease nexin-1 (PN-1) is a potent thrombin inhibitor mainly produced and secreted by exocytosis in astrocytes (Guttridge et al., 1993). Interestingly, NF155 at the paranodal region has a thrombin active site and can be hydrolyzed to NF125 and NF30 by thrombin, which suggests that thrombin may play a fundamental role in regulating the structure and function of nodes of Ranvier (Dutta et al., 2018).

A previous study showed that thrombin and its associated PARs are involved in promoting pain and hyperalgesia (Garcia et al., 2010). As the integrity of the BNB is inseparable from the homeostasis of the peripheral nerve microenvironment, we speculated that compression injury of the TREZ might impair BNB permeability and activate the coagulation system within the TREZ, which would facilitate the peripheral sensitization of TN through specific mechanisms. To validate this hypothesis, several experiments were performed to investigate the effects of compression injury on the BNB of the trigeminal nerve and the structural changes in nodes of Ranvier in the TREZ; the local expression of coagulation/anticoagulation factors was also determined to analyze their potential roles in mediating orofacial mechanical hyperalgesia in a TN rat model.

## Materials and methods

Eighty adult male Sprague–Dawley rats (RRID: MGI:5651135) weighing 140–160 g were obtained from *the Experimental Animal Center of Fujian Medical University* [license no. SCXK(Min)2016–0002]. Rats were housed in a temperature-and humidity-controlled room under a 12:12 h light/dark cycle. Water and food were available *ad libitum*. Rats were randomly assigned to the trigeminal nerve root compression group or the sham operation group. All experiments were approved by the *Animal Care and Use Committee of Fujian Medical University* [approval No. SYXK(Min)2020-0005] on July 17, 2020 and were performed according to the *International Association for the Study of Pain* (IASP) guidelines on using experimental animals to minimize the number of animals used and their suffering. Animal studies were performed in accordance with the ARRIVE guidelines.

### Establishment of the TN animal model

The TN animal model in this study was established as previously described (Luo et al., 2012, 2020; Lin et al., 2018). Briefly, rats were anesthetized with pentobarbital sodium (40 mg/kg, *i.p.*), and a curved skin incision was made above the right eye. Subsequently, the orbital fascia was gently stripped laterally to reveal the infraorbital groove of the maxillary bone to expose the right infraorbital nerve. A small plastic filament (1 mm in diameter) was slowly and carefully inserted into the intracalvarium through the inferior orbital fissure at a depth of 1.2 cm to compress the trigeminal nerve root. Rats in the sham operation group underwent the same procedure without nerve compression. The incision was closed using 6–0 silk sutures.

### Behavioral testing of orofacial mechanical allodynia

**Figure 1:**
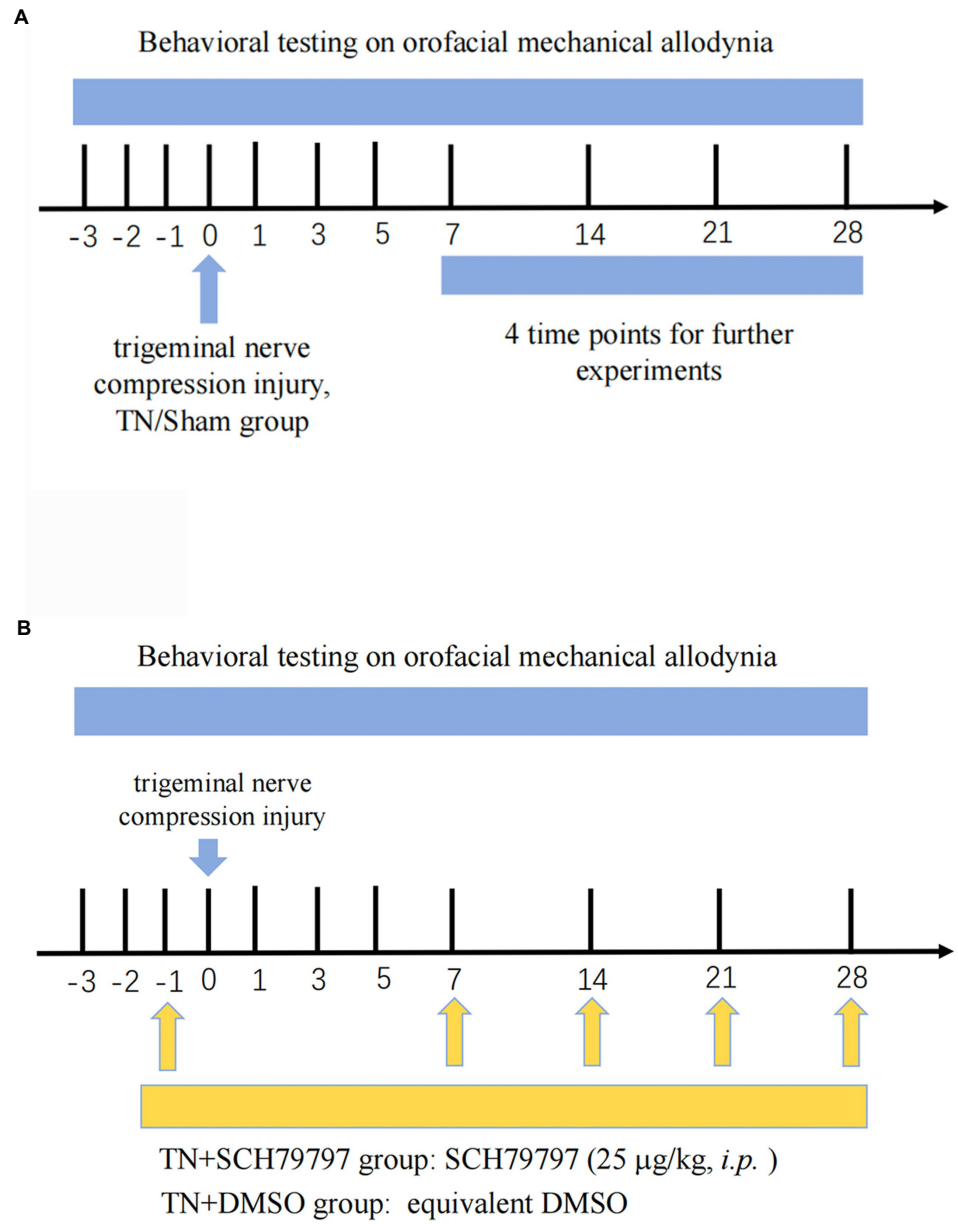
Study timeline. **(A)** Three days prior to the establishment of the TN model, all rats were adapted to the mechanical pain test environment, and the basal orofacial mechanical threshold was tested. Rats randomly received either trigeminal compression injury or sham injury. On PODs 7, 14, 21, and 28, behavioral testing was conducted. Then, the rats were euthanized, and tissues were collected for analysis of BNB integrity, coagulation system-related tests, and structural observation of the nodes of Ranvier. **(B)** Rats underwent the same orofacial mechanical threshold tests. On the day before surgery, the rats randomly received either an intraperitoneal injection of SCH79797 or DMSO. On PODs 7, 14, and 21, rats in the TN + SCH7979 group received SCH7979, while rats in the TN + DMSO group were injected with the same volume of DMSO.

The baseline orofacial mechanical stimulation thresholds of rats were tested before the operation, and after the operation, rats were tested for mechanical allodynia using von Frey filaments (Aesthesio, Ugo Basile, Italy; Figure 1A). All rats were habituated to behavioral testing for 3 days prior to baseline testing. von Frey filaments were applied to the vibrissa pad of the rats to determine the orofacial mechanical allodynia threshold. Each von Frey filament was applied five times. Stimulation always began with the filament that produced the lowest force and was stopped when the threshold was found within the vibrissa pad.

### Evaluation of BNB permeability

On postoperative days (PODs) 7, 14, 21, and 28, rats in the two groups were injected with 2 ml/kg 2% Evans blue (Merck Ca, NJ, United States) through the caudal vein. An hour later, rats in each group (*n* = 3 or 4/group) were anesthetized with sodium pentobarbital (40 mg/kg, *i.p.*) and perfused with cold 0.1 M PBS through the left ventricle to remove intravascular Evans blue. The TREZ was separated and homogenized in 400 μl of formamide. The samples were then incubated at 60°C for 24 h, followed by centrifugation at 10,000 × *g* for 20 min. The optical density value of the supernatant was measured at a wavelength of 620 nm by a microplate reader (BioTek, VT, United States). The extravasation of Evans blue in the tissue was calculated using a standard curve.

Furthermore, Evans blue dye distribution in TREZ tissue sections was observed by confocal microscopy. Rats were anesthetized with sodium pentobarbital (40 mg/kg, *i.p.*) and perfused with 0.1 M PBS. TREZ segments were dissected and cryoprotected with 30% (W/V) sucrose in 0.1 M PBS overnight at 4°C. Trigeminal nerve roots were horizontally sectioned using a cryostat (Leica CM1950, Heidelberger, Germany) to obtain consecutive 10 μm sections. Then, the sections were incubated with 3% bovine serum albumin (BSA) for 30 min to reduce autofluorescence. The extravasated Evans blue-albumin complex yielded red fluorescence in unstained TREZ tissue sections. The processed sections were examined and photographed under a Leica laser confocal microscope (Leica TCS SP8, Heidelberger, Germany).

### Quantitative real-time PCR assay

Rats in both groups (*n* = 6) were deeply anesthetized with sodium pentobarbital (200 mg/kg, *i.p.*) on PODs 7, 14, 21, and 28. Total RNA was extracted in TRIzol reagent (Invitrogen, TX, United States, Cat# 15596018). Subsequently, a High-Capacity cDNA Reverse Transcription Kit (Vazyme, Nanjing, China, Cat# R223) was used to convert RNA into cDNA, while a SYBR-Green Real-Time PCR Kit (Vazyme, Cat# Q311) and a Bio-Rad MiniOption thermocycler (Bio-Rad, CA, United States) were used for detection. Primer sequences are provided in Table 1. The relative expression levels of RNA were evaluated using the 2^−ΔΔCt^ method.

**Table 1:** Oligonucleotide primers used for qRT–PCR.

Gene name	Forward/Reverse (5′–3′)
Prothrombin	(F)GTGAACCTGCCCATTGTAGAG (R)CTCCTCGCTTGGTGTCATTC
PN-1	(F)AAGGTCAGAGAGCCTTCACG (R)TAAACCAGGGAGGCGATGAC
GAPDH	(F)ACGGCAAGTTCAACGGCACAG (R)GAAGACGCCAGTAGACTCCACGAC

### Immunohistochemistry assay

Rats in both groups were deeply anesthetized with sodium pentobarbital (200 mg/kg, *i.p.*) on PODs 7, 14, 21, and 28 and were then perfused through the left ventricle with 4% paraformaldehyde phosphate buffer (pH 7.4). The trigeminal nerve segment from the junction of the trigeminal nerve root with the brainstem to the trigeminal ganglion was dissected and cryoprotected with 30% (W/V) sucrose in 0.1 M PBS overnight at 4°C. Trigeminal nerve roots were horizontally sectioned using a cryostat (Leica CM1950, Heidelberger, Germany) to obtain consecutive 10 μm sections.

For immunohistochemical staining, sections were washed three times in 0.1 M PBS for 10 min and blocked in 3% BSA for 30 min. The sections were subsequently incubated with primary antibodies following the removal of blocking solution without washing. The following primary antibodies were used: rabbit polyclonal anti-Caspr (1:100; Abcam, Cambridge, United Kingdom, Cat# ab34151, RRID: AB_869934), mouse monoclonal anti-myelin basic protein (MBP; 1:1,000; Abcam, Cat# ab62631, RRID: AB_956157), rabbit polyclonal anti-glial fibrillary acidic protein (GFAP; 1:1,000; Proteintech, IL, United States, Cat# 16825-I-AP), and mouse monoclonal anti-PAR1 (1:200; Santa Cruz, Texas, United States, Cat# sc-13,503, RRID: AB_2101175). Biotinylated goat anti-mouse IgG (1:200; Vector, CA, United States, Cat# BA-9200, RRID: AB_2336171), donkey anti-rabbit Alexa Fluor 488 (1:1,000; Invitrogen, CA, United States, Cat# A-21206, RRID: AB_2535792), CyTM3-conjugated streptavidin (1:500; Jackson ImmunoResearch, PA, United States, Cat# 016-160-084, RRID: AB_2337244), and DAPI (1:1,000; Beyotime, Shanghai, China, Cat# C1002) were also used. The immunohistochemical sections were imaged and analyzed with a Leica laser confocal microscope.

### Western blotting assay

Rats in the TN and sham groups were sacrificed after anesthetization with sodium pentobarbital (200 mg/kg, *i.p.*), after which the trigeminal nerve root was quickly removed and rapidly frozen in liquid nitrogen. The samples from different groups were homogenized in extraction buffer (100 mM Tris, pH 7.4) containing 2 mM phenylmethanesulfonylfluoride and 10 mg/ml aprotinin. The solutions were centrifuged at 12,000 × *g* for 10 min at 4°C. The protein concentration of the supernatants was determined using a BCA Protein Assay Kit (Beyotime, Cat# P0010). Protein samples (10–20 μg) were loaded on 8 or 10% polyacrylamide gels for SDS–PAGE and were then transferred onto polyvinylidene difluoride membranes. The membranes were blocked with 5% nonfat milk in Tris-buffered saline containing 0.1% Tween 20 for 1 h at room temperature and incubated with the following primary antibodies overnight at 4°C: rabbit monoclonal anti-NF155 (1:1,000; Cell Signaling Technology, MA, United States, Cat# 15035, RRID: AB_2798693), mouse monoclonal anti-PAR1 (1:500; Santa Cruz, Cat# sc-13,503, RRID: AB_2101175), mouse polyclonal anti-*β*-tubulin (1:10,000; Bioworld, MN, United States, Cat# BS1482M), and rabbit polyclonal anti-GAPDH (1:10,000; Bioworld, Cat# AP0066, RRID: AB_2797448). Then, the tissues were incubated with goat anti-mouse IgG (H + L)-HRP (1:5,000; Bioworld, Cat# BS12478, RRID: AB_2773727) or goat anti-rabbit IgG (H + L)-HRP (1:10,000, Bioworld, Cat# BS13278, RRID: AB_2773728) secondary antibody for 2 h at room temperature. The bands were detected using Immobilon Western Chemiluminescent reagent (Bio-Rad, CA, United States).

### Administration of the PAR1 antagonist SCH79797

The PAR1 antagonist SCH79797 (25 μg/kg; Abcam, Cat# ab120858) was selected for *in vivo* intervention experiments (Figure 1B). SCH79797 was dissolved in dimethyl sulfoxide (DMSO) and diluted in sterile 0.01 M PBS. Rats were randomly divided into four groups: the sham group, TN group, TN + SCH79797 group, and the TN + DMSO group. Rats in the TN + SCH79797 and TN + DMSO groups were given an intraperitoneal injection of SCH79797 (25 μg/kg) and an equivalent volume of DMSO, respectively. After testing the mechanical pain thresholds on PODs 7, 14, and 21, rats in the TN + SCH79797 and TN + DMSO groups were repeatedly injected with SCH79797 and DMSO, respectively, once a week.

### Statistical analysis

All data presented represent the mean of at least three replicates of independent samples. Data are presented as the mean ± SD and were analyzed using two-way ANOVA and Sidak’s multiple comparisons test. Statistical analysis and graphing were performed using R language and GraphPad Prism v8.0 (GraphPad Software, Inc., CA, United States). Significant differences are denoted by asterisks, and *p* < 0.05 was considered statistically significant.

## Results

### TREZ compression injury induced TN and destroyed the BNB permeability

**Figure 2:**
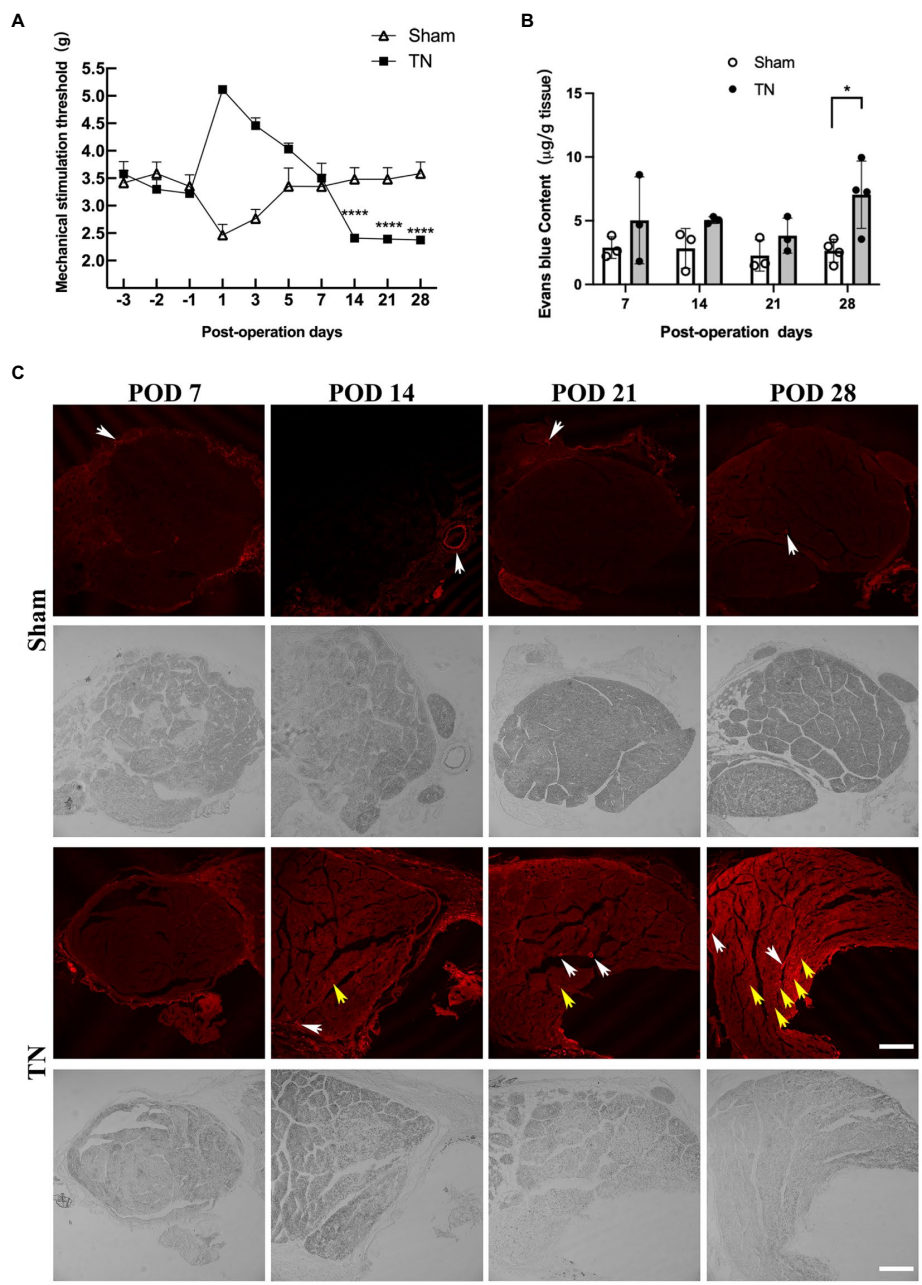
Mechanical compression injury of the trigeminal nerve induced orofacial allodynia and disruption of the blood-nerve barrier. **(A)** Mechanical compression injury of the trigeminal nerve induced changes in the orofacial mechanical stimulation thresholds. Compared with the sham group, TN group rats were insensitive to the mechanical stimulation after TREZ compression injury surgery to POD 7, but they displayed a significant mechanical allodynia according to the withdrawal response of TN rats from POD 14 to 28. (mean ± SD, *n* = 5, *n* indicates the number of independent animals; ns: no significant difference, ^****^p < 0.0001; two-way ANOVA). **(B)** The content of Evans blue in TREZ tissue was tested in both groups. Compared with the sham group, the content of Evans blue in the TREZ of TN group was significantly increased on POD 28 (mean ± SD, *n* = 3 or 4/group, ^*^p < 0.05; two-way ANOVA). **(C)** The exudation of Evans blue (red) in the TREZ in the sham group and the TN group was observed with a Leica TCS SP8 X laser confocal microscope, bar = 250 μm.

According to the results of orofacial mechanical behavioral tests in the sham and TN groups, no significant difference was observed in the baseline mechanical pain thresholds between the two groups (*F*_1,27_ = 1.64, *p* > 0.05; Figure 2A). Interestingly, the mechanical pain thresholds of rats in the sham group dramatically declined on POD 1 and then gradually increased and approached baseline levels from PODs 3 to 7. However, rats in the TN group exhibited a converse trend of mechanical pain thresholds, i.e., the thresholds markedly increased and peaked on POD 1 and then gradually decreased and approached the baseline levels from PODs 3 to 7. In addition, the mechanical pain thresholds on PODs 14, 21, and 28 in the sham group were maintained at baseline levels and were significantly higher than those in the TN group. These results indicate that acute injury following the operation transiently facilitated orofacial mechanical allodynia in the sham group but caused temporary orofacial numbness in the TN group. Rats in the TN group exhibited obvious orofacial mechanical allodynia on PODs 14, 21, and 28, which suggests that the TN animal model induced by chronic compression of the trigeminal root was successfully constructed.

To examine whether compression injury of the TREZ induces disruption of the BNB, an Evans blue extravasation test was conducted. The results showed that the permeability of the BNB increased slightly in the TN group on PODs 7, 14, and 21 and was markedly upregulated on POD 28 compared with the sham group (Figures 2B,C). Notably, Evans blue exudation in the sham group was mainly restricted to the vascular wall and surrounding area (Figure 2C, white arrow), while endoneurial exudation was not obvious. In contrast, more Evans blue exudation was observed in the nerve bundle (Figure 2C and Supplementary Figure 1, yellow arrow) in the TN group, which indicated that the integrity of the BNB was impaired upon compression injury of the TREZ.

### Chronic compression caused structural changes at nodes of Ranvier in the TREZ

**Figure 3:**
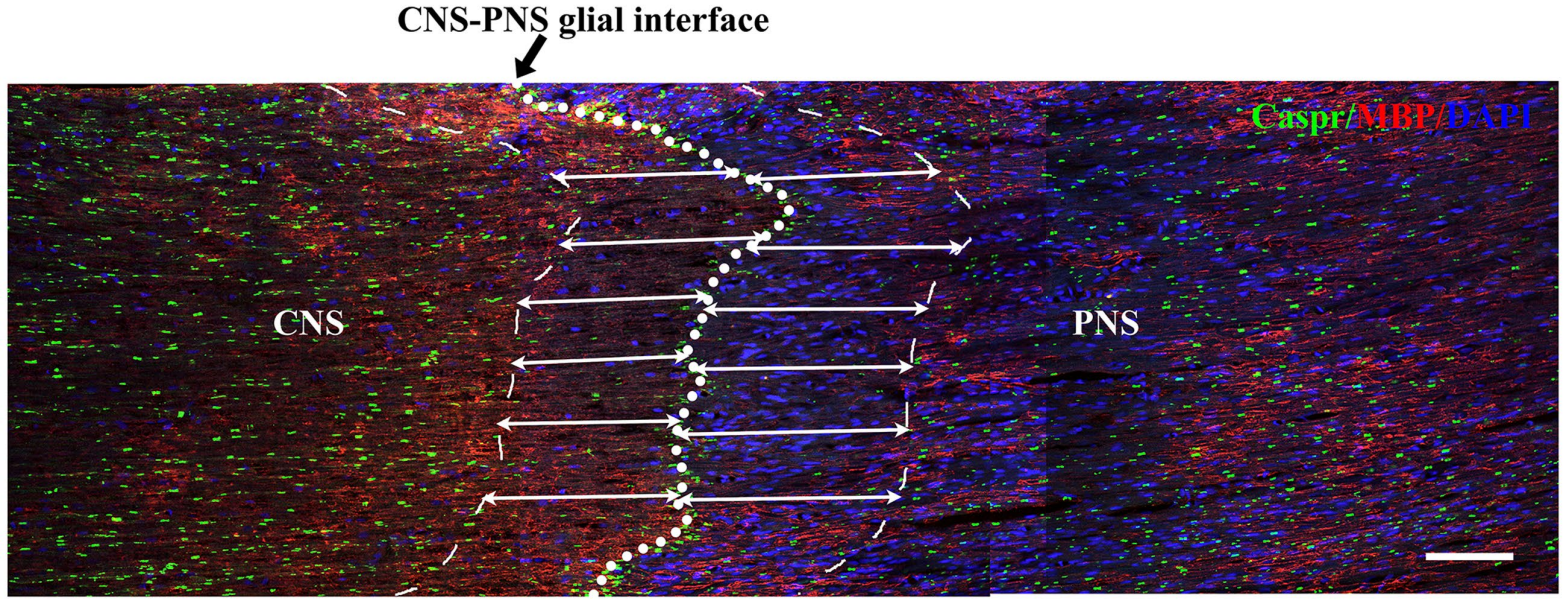
Distribution of nodes of Ranvier in the TREZ of rats. MBP (red) was mainly distributed in oligodendrocytes in the CNS, while a small amount was expressed in some Schwann cells in the PNS. Caspr (green) immunofluorescence staining showed the paranodal region of myelinated nerve fibers. The white circle dotted line (black arrow) indicates the CNS-PNS interface, where the nodes of Ranvier were highly concentrated, while the thin dotted lines show the border of node-depleted zones on both sides. The straight lines with arrows represent the distance from the boundary of the node-depleted zones to the CNS-PNS interface. Bar = 100 μm. Logical size, *X* = 2048 pixels, *Y* = 2048 pixels, *Z* = 1 pixel, pixel size = 0.284 μm.

**Figure 4:**
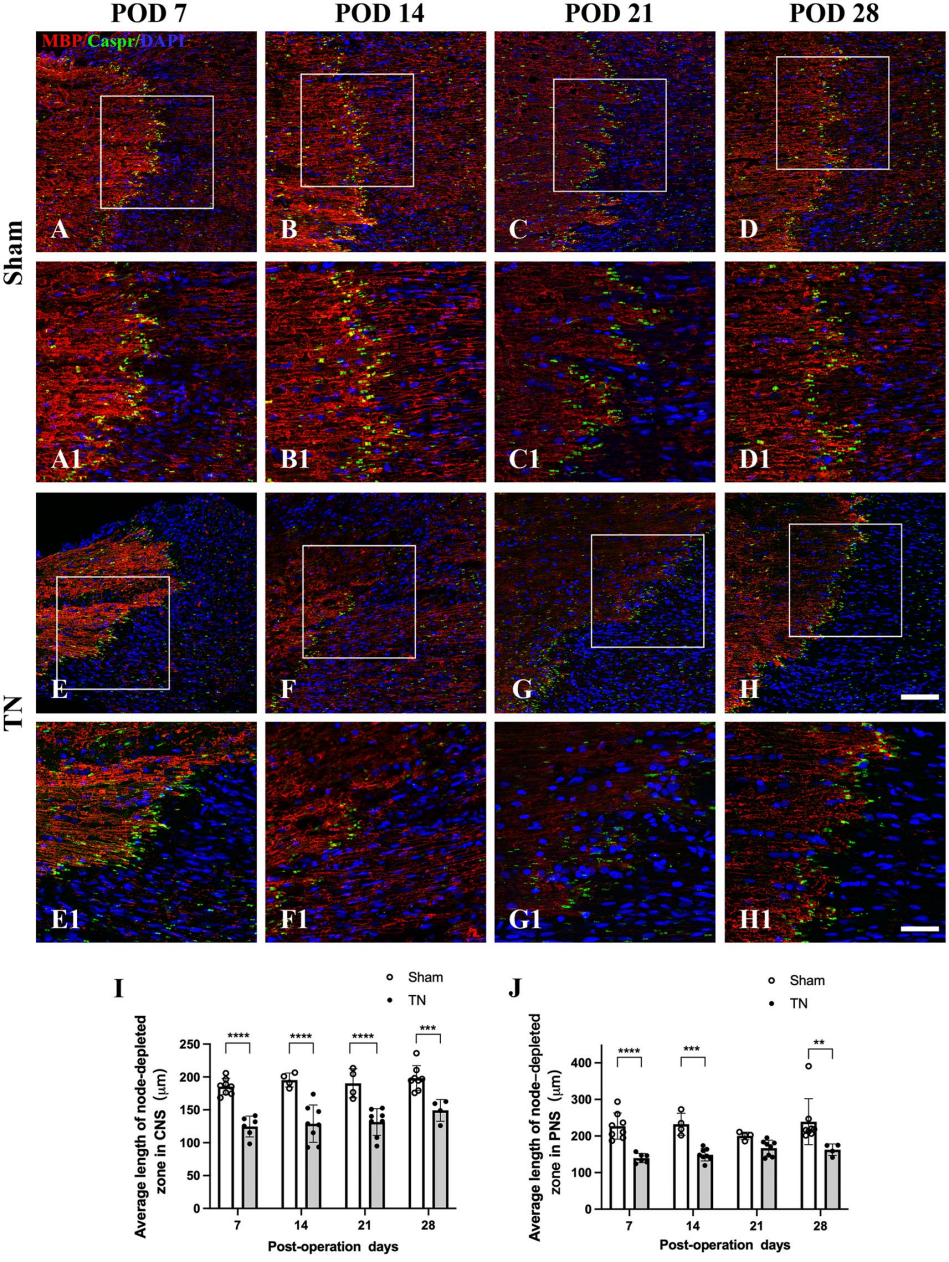
Changes in node-depleted zones in the TREZ. Caspr (green) was used to show the paranodal region of myelinated nerve fibers, while MBP (red) staining showed the myelin sheaths in both the CNS and PNS. **(A–D, A1–D1)** The nodes of Ranvier were highly concentrated at the CNS-PNS interface of rats in the sham group, with obvious node-depleted zones in both the CNS and PNS. **(E–H, E1–H1)** The nodes of Ranvier at the CNS-PNS interface of rats in the TN group were slightly chaotic, and the myelin sheaths were partially destroyed. **(A–H)** Bar = 100 μm, images were obtained using the same settings (logical size, *X* = 2048 pixels, *Y* = 2048 pixels, *Z* = 1 pixel, pixel size = 0.284 μm). **(A1–H1)** Bar = 50 μm, images were obtained using the following settings: logical size, *X* = 2048 pixels, *Y* = 2048 pixels, *Z* = 1 pixel, pixel size = 0.114 μm. **(I–J)** A Leica TCS SP8 X laser confocal microscope was used to measure the length of node-depleted zones. All images were obtained using the same settings (logical size, *X* = 2048 pixels, *Y* = 2048 pixels, *Z* = 1 pixel, pixel size = 0.114 μm). Compared with the sham group, TN group rats had shorter lengths of the node-depleted zone in both CNS and PNS side. Data are expressed as the mean ± SD and were analyzed using two-way ANOVA followed by Sidak’s multiple comparisons test. ^**^p < 0.01, ^***^p < 0.001, ^****^p < 0.0001.

**Figure 5:**
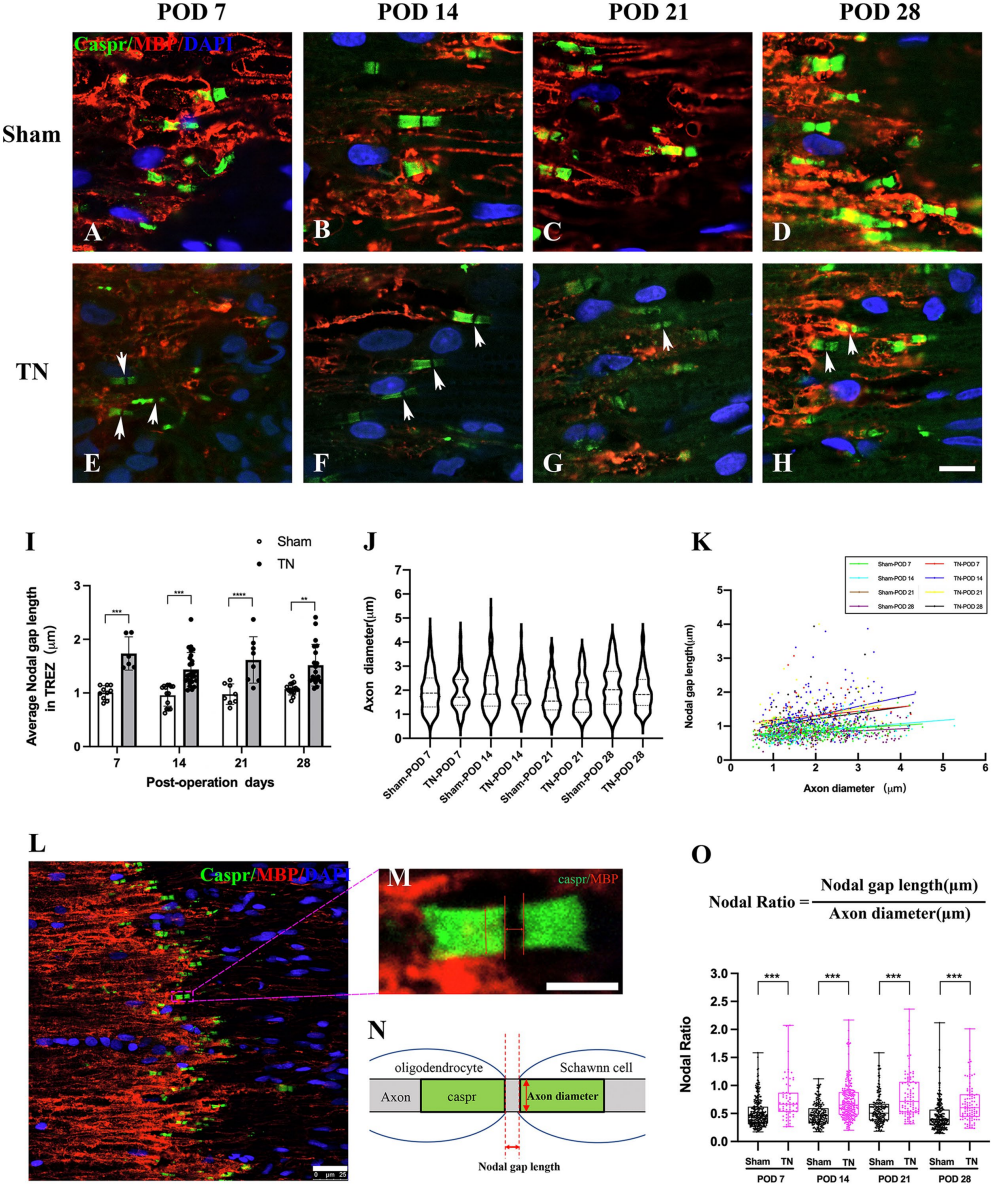
Changes in nodal gap lengths, axon diameters and nodal ratios at the CNS-PNS interface in the TREZ. **(A–H)** Magnified observations of the CNS-PNS interface in the TREZ. MBP (red) immunofluorescence staining is mainly distributed in oligodendrocytes on the CNS side, while a small amount can also be expressed in Schwann cells in the PNS. Caspr (green) immunofluorescence staining showed paranodal regions and nodal gaps (white arrows). Bar = 10 μm. Images were obtained using the same Leica TCS SP8 microscope settings (logical size, *X* = 2048 pixels, *Y* = 2048 pixels, *Z* = 1 pixel, pixel size = 0.284 μm). **(I)** The nodal gap lengths at the CNS-PNS interface in the TREZ in the TN group were longer than those in the sham group (*p < 0.001*). Data are expressed as the mean ± SD and were analyzed using two-way ANOVA followed by Sidak’s multiple comparisons test. ^**^p < 0.01, ^***^p < 0.001, ^****^p < 0.0001. **(J)** No significant differences were observed in the axon diameters of myelinated nerve fibers at the CNS-PNS interface between the two groups. **(K)** The distribution of myelinated nerve fibers with different diameters and nodal gap lengths at the CNS-PNS interface in the TREZ. Linear correlation regression analysis showed that in the TN group, larger diameter myelinated nerve fibers had a wider nodal gap at the CNS-PNS interface. **(L–N)** Images of nodes of Ranvier at the CNS-PNS interface in the TREZ and theFIGURE 5 Continuedcorresponding schematic diagram. L: bar = 25 μm. M: bar = 5 μm. **(O)** From PODs 7 to 28, the nodal ratios in the TN group were significantly larger than those in the sham group. Box plot shows all points (Min to Max) and were analyzed using two-way ANOVA followed by Sidak’s multiple comparisons test. ^***^p < 0.001.

Double immunofluorescence staining for Caspr and MBP allowed clear visualization of the CNS-PNS interface in the TREZ (Figure 3). A higher density of Caspr protein was observed in the TREZ (Figures 3, 4A1–H1), and two adjacent Caspr protein expression regions were used to measure the gap between the nodes of Ranvier (Figures 5A–H). In line with previous reports (Henry et al., 2005), two virtually node-free domains were seen, i.e., node-depleted zones were observed flanking the CNS-PNS interface (Figure 3). For further statistical analysis, Leica TCS SP 8 confocal microscopy with ultrahigh resolution was used. To measure the length of node-depleted zones, images of 2,048 × 2,048 × 1 pixels of TREZ were obtained (pixel size = 0.284 μm) and six lines were selected for measuring (Supplementary Figure 2). To measure the diameter of the nerve fibers and nodal gap length at the CNS-PNS interface, images were obtained at higher magnification (logical size, X = 2048 pixels, Y = 2048 pixels, Z = 1 pixel, and pixel size = 0.114 μm). All images were measured and analyzed by a blinded expert. We also calculated and analyzed the nodal ratio, which is the ratio of nodal gap length to the axon diameter, as previously described (Tavares et al., 2019; Figures 4A–H). Our results showed that the length of the node-depleted zones in the TN group was shorter than that in the sham group (Figures 4I,J), while the average lengths of nodal gaps at the CNS-PNS interface in the TN group were significantly wider than those in the sham group (Figure 5I). No difference was found in axon diameters between the two groups (Figure 5J). However, the nodal ratio in the TN group was significantly increased, and further linear correlation regression analysis showed that in the TN group, larger diameter myelinated nerve fibers had a wider nodal gap at the CNS-PNS interface (Figures 5K–O). These results indicated chronic compression caused distinct structural changes at nodes of Ranvier in the TREZ.

### Chronic compression injury resulted in upregulation of prothrombin/PN-1 and hydrolysis of paranodal protein NF155 in the TREZ

**Figure 6:**
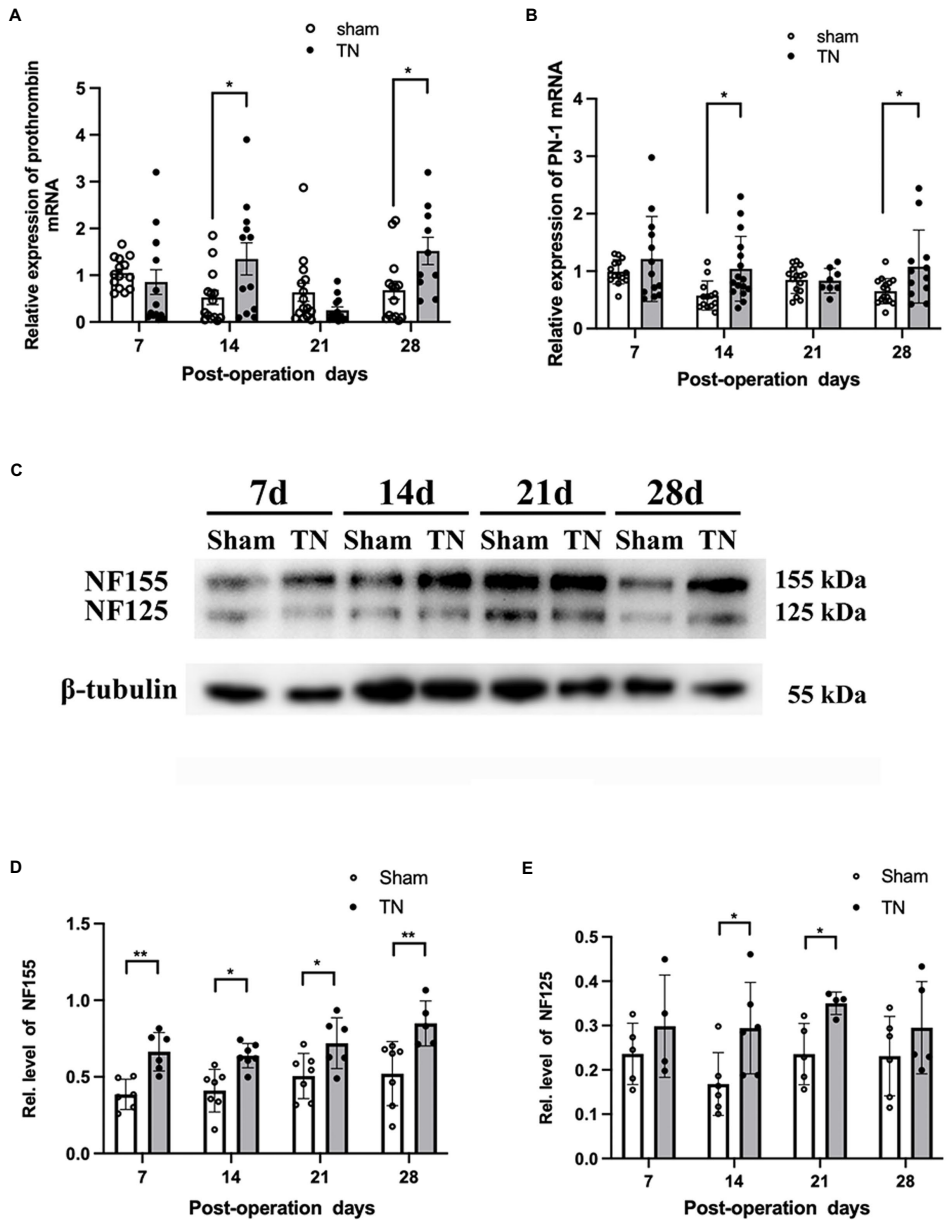
Chronic compression injury changed the expression of prothrombin, PN-1, NF155, and NF125 in the TREZ. **(A)** Transcription levels of prothrombin in the TREZ were significantly increased in the TN group on PODs 14 and 28 compared with the sham group. **(B)** Transcription levels of PN-1 in the TN group were significantly upregulated on PODs 14 and 28. **(C)** Two protein bands representing NF155 and NF125 were detected in the immunoblotting assay. **(D)** Compared with the sham group, the expression of NF155 in the TREZ of the TN group increased significantly from PODs 7 to 28. **(E)** The expression of NF125 was higher in the TN group on PODs 14 and 21 than in the sham group. Data are presented as the mean ± SD (*n* = 4–7/group). All comparisons were analyzed using two-way ANOVA and Sidak’s multiple comparisons test. ^*^p < 0.05, ^**^p < 0.01, ^***^p < 0.001, and ^****^p < 0.0001.

To determine if the coagulation/anti-coagulation system in the TREZ was activated during the process of TN, the expression levels of prothrombin and PN-1 were measured by quantitative real-time PCR (qRT–PCR). According to qRT–PCR, no differences were observed in the transcription level of prothrombin between the two groups on PODs 7 and 21, while the transcription level of prothrombin in the TN group was significantly increased compared with that in the sham group on PODs 14 and 28 (Figure 6A). In addition, the relative expression of PN-1 mRNA in the TN group was significantly upregulated on PODs 14 and 28 compared with that in the sham group (Figure 6B).

Due to the activation of the coagulation/anti-coagulation system in the TREZ, we speculated the paranodal protein NF155 in the TREZ may undergo proteolysis during TN pathogenesis, to validate the hypothesis; a Western blotting assay was performed. As shown by Western blotting, two bands of NF155 protein (155 and 125 kDa) were detected (Figure 6C), which suggests that some NF155 proteins may have been hydrolyzed into NF125 and NF30. The expression of NF155 protein in the TN group was upregulated compared with that in the sham group (Figure 6D). Additionally, the protein expression of NF125 in the TREZ in the TN group was increased on PODs 14 and 21, but the difference between the two groups on POD 28 was not significant (Figure 6E).

### Inhibition of PAR1 with the antagonist SCH79797 ameliorated mechanical allodynia induced by compression injury of the TREZ

**Figure 7:**
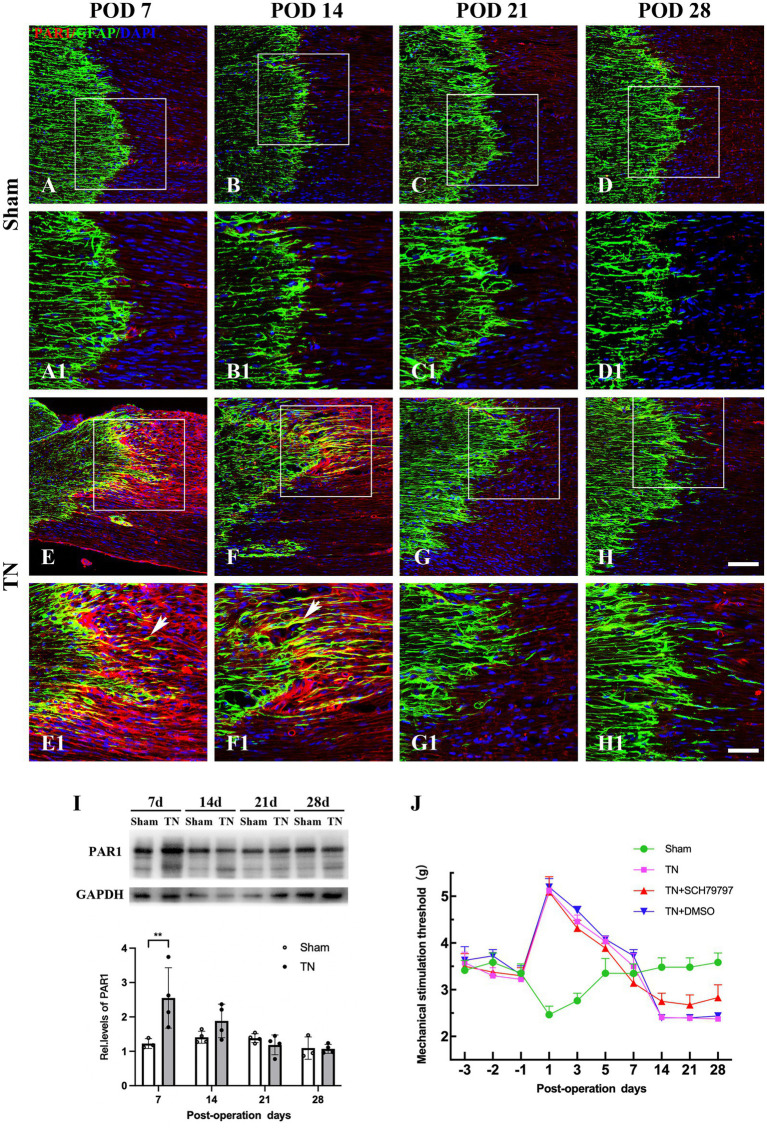
Changes in the expression and distribution of PAR1 in the TREZ of rats. **(A–D)** PAR1 (red) was expressed at low levels in the TREZ in the sham group. **(E–H)** PAR1 (red) expression in rats in the TN group was increased compared with that in rats in the sham group. Some PAR1 was expressed in GFAP-positive astrocytes (white arrows). **(A–H)** Bar = 100 μm, A1-H1: bar = 50 μm. **(I)** On POD 7, the expression of PAR1 protein in the TREZ of rats in the TN group was significantly higher than that in rats in the sham group. On PODs 14, 21, and 28, PAR1 expression in the TN group gradually decreased until it was similar to that in the sham group. Data are expressed as the mean ± SD (*n* = 3 or 4/group) and were analyzed using two-way ANOVA followed by Sidak’s multiple comparisons test. ^**^p < 0.01. **(J)** The orofacial mechanical stimulation thresholds of rats in different groups. In contrast to the sham group, rats in the other three groups all displayed a markedly higher orofacial mechanical pain threshold on POD 1, which FIGURE 7 Continuedthen gradually decreased to the baseline thresholds from PODs 3 to 7. Rats in the TN + SCH79797 group exhibited higher mechanical thresholds than those in the TN and TN + DMSO groups from POD 14, especially on PODs 14 and 28. Data are expressed as the mean ± SD (*n* = 4 or 5/group).

To define the spatiotemporal expression pattern of PAR1 in the TREZ of the TN rats, an immunohistochemistry assay was performed. Immunofluorescence staining showed that PAR1 was expressed on both sides of the TREZ transitional zone and was accompanied by GFAP-immunopositive astrocytes that extended from the central to the peripheral side of the TREZ (Figures 7A-H). Subsequent immunoblotting assays revealed that PAR1 protein expression in the TREZ in the TN group was significantly higher than that in the sham group on POD 7 and then gradually decreased to a level similar to that of the sham group (Figure 7I).

To further analyze the effect of PAR1 on orofacial hyperalgesia, the PAR1 antagonist SCH79797 was administered to TN rats. According to the behavioral test results, rats in all groups had similar baseline orofacial mechanical pain thresholds before surgery. In contrast to the sham group, rats in the other three groups all displayed a markedly higher orofacial mechanical pain threshold on POD 1, which then gradually decreased to the baseline thresholds from PODs 3 to 7. However, the mechanical pain thresholds of the sham group showed a trend opposite that of the other three groups, i.e., they declined to the nadir on POD 1, then gradually increased and approached the baseline values from PODs 3 to 7, which was similar to the behavior test results described above. Notably, on PODs 14, 21, and 28, the mechanical pain thresholds of the sham group were maintained at baseline values and were higher than those of the other three groups (*F*_3,48_ = 172.8, *p* < 0.0001; Figure 7J). In addition, rats in the TN + SCH79797 group exhibited higher mechanical pain thresholds than those in the TN and TN + DMSO groups from POD 14, especially on PODs 14 and 28 (*F*_2,33_ = 10.54, *p* < 0.0001; Figure 7J). These results indicate that SCH79797 ameliorated orofacial mechanical allodynia induced by compression injury of the TREZ.

## Discussion

Blood-nerve barrier is a special barrier consisting of an endoneurial capillary barrier and a perineurial barrier (Hirakawa et al., 2003; Weerasuriya and Mizisin, 2011). It was reported that partial sciatic nerve ligation injury induces an increase in the permeability of the BNB and local inflammation (Lim et al., 2014). In addition, disruption of the BNB allows the infiltration of immunocytes and promotes neuroinflammation, which leads to the progression of nerve sensitization and neuropathic pain development after nerve injury (Moreau et al., 2016). However, whether compression injury of the trigeminal nerve could induce impairment of the BNB and the way in which these concomitant effects contribute to orofacial mechanical allodynia in TN is unclear.

Evans blue is widely used as a vascular permeability marker for evaluating the integrity of the blood-tissue barrier (Radu and Chernoff, 2013; Ahishali and Kaya, 2021). In the present study, we observed that Evans blue notably exuded around the nerve bundle membrane and endoneurium in the TREZ after compression injury, which suggests that chronic compression of the TREZ impaired the integrity of the BNB and markedly increased its permeability.

A previous study showed that chronic compression of the trigeminal nerve induces changes in plasticity in various glial cells in the TREZ (Luo et al., 2019). In that study, upon chronic compression injury, GFAP-positive astrocytes on the CNS side of the TREZ were activated, and their protrusions markedly extended to the peripheral side. In addition, myelin basic protein (MBP) and p75^NGF^ expression was decreased significantly in the early stage of compression injury, which suggests the occurrence of nerve demyelination in the TREZ during the pathogenesis of TN (Luo et al., 2019). The nodes of Ranvier are myelin-free regions that are critical for the saltatory conduction of electrical signals along myelinated nerve fibers. The partitioned connections generated by myelin-forming glial cells and the axon membrane restrict sodium ion channels to the nodes of Ranvier and the recruitment of potassium ions to the paranodal region (Rasband and Peles, 2021). Growing evidence suggests that changes in the distribution of ion channels around the nodes of Ranvier could affect the signal conduction of nerve fibers and the connections between neurons (Nelson and Jenkins, 2017; Faivre-Sarrailh, 2020). Here, we discovered that compression injury of the TREZ induced structural changes in local nodes of Ranvier, which were characterized by a widened nodal gap length and a shortened length of the node-depleted zone. As the length of the nodal gap and the distance between the nodes can directly affect the conduction speed of the myelinated nerve fibers (Arancibia-Carcamo et al., 2017; Tavares et al., 2019), we speculated that the structural changes in the nodes of Ranvier in the TREZ might modulate the sensitivity of trigeminal afferent nerves, which may contribute to orofacial mechanical hyperalgesia in TN.

Thrombin is a trypsin-like allosteric serine protease activated within the coagulation cascade that is formed after cleavage of its precursor prothrombin by activated Factor X (FXa). A previous study reported that in the case of blood–brain barrier (BBB) breakdown, thrombin enters the brain and reaches high concentrations (Sokolova and Reiser, 2008), which indicates that damage to the BNB may also be an important cause of the imbalance of the coagulation and anticoagulation system. In addition to its central role in the coagulation cascade, the generation of thrombin leads to receptor-mediated inflammatory responses, cell proliferation/modulation, cell protection, and apoptosis in a concentration-dependent manner. Several groups confirmed that low concentrations of thrombin could cause neuron and astrocyte modifications, induce glial cell proliferation, and exert neuroprotective effects, while high concentrations of thrombin exert neurotoxic effects with disruption of the BBB, edema, and inflammation (Dihanich et al., 1991; Krenzlin et al., 2016). PN-1 is mainly produced and secreted by astrocytes *via* exocytosis (Gofrit and Shavit-Stein, 2019) and is the primary endogenous serine protease inhibitor in the nervous system that inactivates serine proteases, including thrombin (Beilin et al., 2005). In this study, the mRNA expression of prothrombin and PN-1 in the TREZ was consistently upregulated in the TN group, which indicates that compression injury induced the local activation of coagulation and anticoagulation factors; this in turn may affect the homeostasis of the TREZ microenvironment in a thrombin-dependent manner.

A recent study demonstrated that NF155 in the paranodal region contains a thrombin active site and can be hydrolyzed to NF125 and NF30 by thrombin. Therefore, thrombin may play an essential role in regulating the structure and function of nodes of Ranvier (Dutta et al., 2018). Similarly, our study revealed that compression injury of the trigeminal nerve induced the proteolysis of NF155 in the TREZ, which may be mediated by activated thrombin, and eventually caused the dissociation of Caspr and NF155.

Protease-activated receptor 1, the major receptor for thrombin, is located at synapses and at the nodes of Ranvier (Chapman, 2013). Growing evidence has shown that thrombin acts *via* its receptors and participates in the processes of nerve injury and allodynia (Dong et al., 2013; Smith and Winkelstein, 2017), but whether thrombin-PAR1 is involved in TN induced by compression injury of the TREZ is unclear. It was reported that neuron-generated thrombin may lead to astrocyte activation and paracrine neuroprotection through PAR1 (Rajput et al., 2020). Several studies have demonstrated that PAR1 activation modulates the form and function of astrocytes (Vance et al., 2015; Serwanski et al., 2017; Yoon et al., 2018). In this study, PAR1 expression was increased in the TREZ at an early stage of compression injury. Double immunofluorescence staining confirmed the colocalization of PAR1 and GFAP in the central part of the TREZ, which suggests that PAR1 might mediate astrocyte activation during the pathogenesis of TN. A previous study reported that perinodal astrocytes regulate the proteolysis of NF155 by thrombin *via* PN-1 secretion (Berkowitz et al., 2021). In addition, a variety of glial cells, including astrocytes, normally insert themselves into the nodes of Ranvier to regulate structural plasticity and signal conduction (Serwanski et al., 2017; Jha et al., 2019; Li et al., 2020). Based on the above findings, we assumed that PAR1-activated glial cells of the TREZ may play a critical role in modulating orofacial mechanical hyperalgesia in TN by affecting the structure of nodes of Ranvier. To validate our hypothesis, chemical inhibitors were administered to block PAR1, and the results showed that the antagonist SCH79797 notably shortened the recovery from postoperative pain and relieved the pain sensitization caused by compression injury, which suggests that PAR1 mediated orofacial mechanical hyperalgesia in our TN rat model.

In this study, immunohistochemical staining and laser confocal microscopy were used to measure the length of the depletion zones of the nodes of Ranvier in the TREZ region, the diameter of nerve fibers and the length of the nodal gap, which contributed to a better understanding of the potential meaning of the special anatomical structure of the nodes of Ranvier in the TREZ region during TN pathogenesis. However, there also exist some limitations. For example, as the border of the node-depleted zone is irregular, a certain degree of subjectivity is inevitable when the measurement was conducted. Independent repeated measurements by the blind observers would help to minimize the statistical variability. Moreover, immunohistochemistry methods might not the best way to exhibit the ultrastructures of nodes of Ranvier in the TREZ, as the resolution of the images, thick of the fibers may both influenced the observation results. In addition, although the depletion zones of the nodes of Ranvier were shortened in the TREZ upon chronic compression injury, the underlying mechanisms and their potential roles are still unknown.

In summary, chronic compression injury of the TREZ in this TN animal model destroys and increases the permeability of the BNB and results in higher prothrombin and PN-1 expression. Activated thrombin may then directly regulate myelin-axon connections at the nodes of Ranvier in the TREZ transitional zone or activate astrocytes through PAR1 to affect the structure of the nodes of Ranvier. These results indicate that BNB disruption and coagulation system activation may be involved in the peripheral sensitization in trigeminal neuralgia.

## Data availability statement

The raw data supporting the conclusions of this article will be made available by the authors, without undue reservation.

## Ethics statement

The animal study was reviewed and approved by Animal Care and Use Committee of Fujian Medical University [approval no. SYXK(Min)2020-0005] on July 17, 2020.

## Author contributions

L-XZ and S-WL performed the experiments and analyzed the data. R-HQ participated in animal model building and behavioral testing. LL was responsible for confocal imaging. Y-FG and FW helped with data analysis. FW, D-SL, and Y-QL designed the experiments and wrote and revised the manuscript. All authors contributed to the article and approved the submitted version.

## Funding

This work was funded by the National Natural Science Foundation of China (grant number: 82171213) and Startup Fund for scientific research, Fujian Medical University (grant number: 2021QH2005).

## Conflict of interest

The authors declare that the research was conducted in the absence of any commercial or financial relationships that could be construed as a potential conflict of interest.

## Publisher’s note

All claims expressed in this article are solely those of the authors and do not necessarily represent those of their affiliated organizations, or those of the publisher, the editors and the reviewers. Any product that may be evaluated in this article, or claim that may be made by its manufacturer, is not guaranteed or endorsed by the publisher.

## Supplementary material

Supplementary Figure 1 Mechanical compression injury of the trigeminal nerve induced disruption of the blood-nerve barrier. Evans blue exudation in the sham group was mainly restricted in the vascular wall and surrounding area (white arrow), while the endoneurial exudation was not obvious. In contrast, more Evans blue exudation was observed in the nerve bundle (yellow arrow) in the TN group.

Supplementary Figure 2 Diagram of TREZ area structure. The black dotted line represents the highly concentrated nodes of Ranvier at the CNS-PNS glial interface in the TREZ. The pink dotted lines represent the border of the node-depleted zone in CNS and PNS, respectively. Yellow area represents the node-depleted zones in CNS, while dark gray area represents the node-depleted zones in PNS. Blue box represents the areas photographed by the Leica TCS SP8 confocal microscope. Images were taken with same logical size (*X* = 2048 pixels, *Y* = 2048 pixels, Z = 1 pixel). To measure the length of node-depleted zones, every pixel length represents physical 0.284 μm. The six lines (Red) paralleled to the longitudinal axis was selected and measured by a blind expert. The mean of these lines were used for evaluating the length of the node-depleted zone.
