# Supplementary figures and images for: Blood-nerve barrier disruption and coagulation system activation induced by mechanical compression injury participate in the peripheral sensitization of trigeminal neuralgia
Source: Front Mol Neurosci. 2022 Dec 20;15:1059980. doi: 10.3389/fnmol.2022.1059980 (PMC9810503; doi:10.3389/fnmol.2022.1059980)

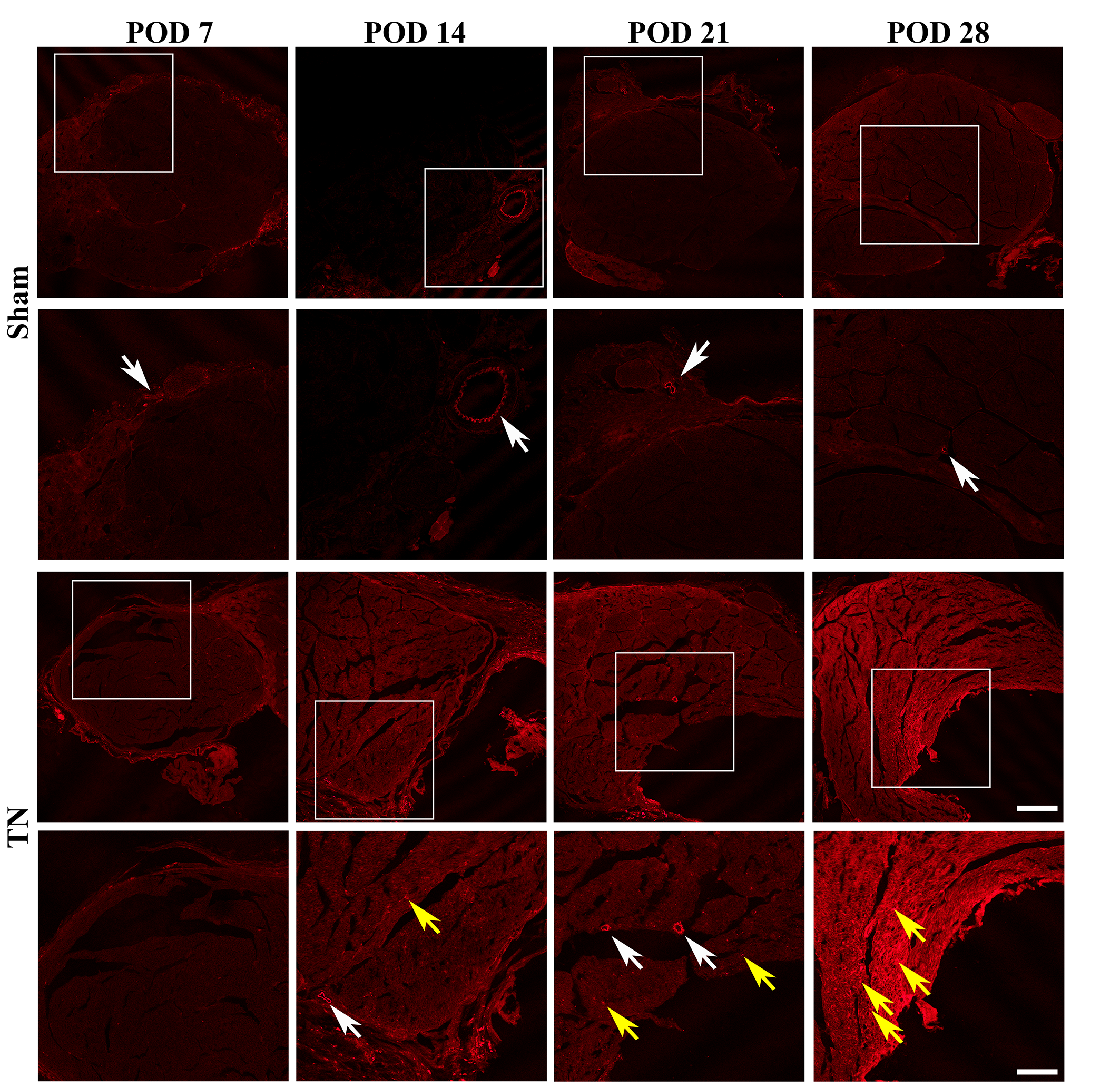

Supplement: Supplementary Figure 1 — Mechanical compression injury of the trigeminal nerve induced disruption of the blood-nerve barrier. Evans blue exudation in the sham group was mainly restricted in the vascular wall and surrounding area (white arrow), while the endoneurial exudation was not obvious. In contrast, more Evans blue exudation was observed in the nerve bundle (yellow arrow) in the TN group. [file Image_1.TIF]

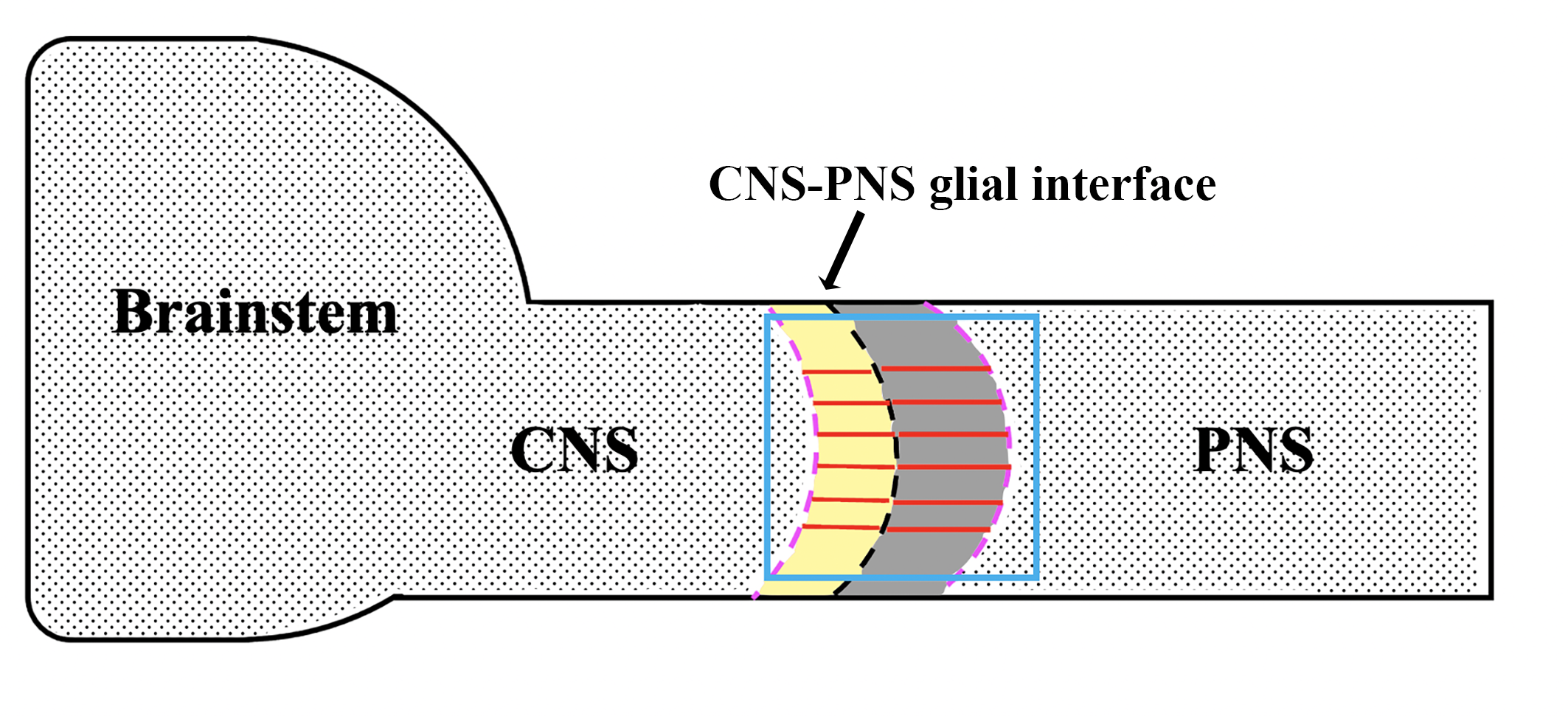

Supplement: Supplementary Figure 2 — Diagram of TREZ area structure. The black dotted line represents the highly concentrated nodes of Ranvier at the CNS-PNS glial interface in the TREZ. The pink dotted lines represent the border of the node-depleted zone in CNS and PNS, respectively. Yellow area represents the node-depleted zones in CNS, while dark gray area represents the node-depleted zones in PNS. Blue box represents the areas photographed by the Leica TCS SP8 confocal microscope. Images were taken with same logical size (X = 2048 pixels, Y = 2048 pixels, Z = 1 pixel). To measure the length of node-depleted zones, every pixel length represents physical 0.284 μm. The six lines (Red) paralleled to the longitudinal axis was selected and measured by a blind expert. The mean of these lines were used for evaluating the length of the node-depleted zone. [file Image_2.TIF]
